# *Juniperus sabina* L. as a Source of Podophyllotoxins: Extraction Optimization and Anticholinesterase Activities

**DOI:** 10.3390/ijms231810205

**Published:** 2022-09-06

**Authors:** Shengnan Xu, Xinru Li, Shi Liu, Peilin Tian, Dengwu Li

**Affiliations:** 1 College of Forestry, Northwest A & F University, Xianyang 712100, China; 2 Shaanxi Key Laboratory of Economic Plant Resources Development and Utilization, Xianyang 712100, China

**Keywords:** homogenization-assisted ultrasonic extraction, *Juniperus sabina* L., podophyllotoxins, anticholinesterase activity

## Abstract

*Juniperus sabina* L. (*J. sabina*) has been an important plant in traditional medicine since ancient times. Its needles are rich in podophyllotoxin, a precursor compound to anti-tumor drugs. However, no systematic research has been done on *J. sabina* as a source of podophyllotoxins or their biological action. Hence, extracts of podophyllotoxin and deoxypodophyllotoxin were the main optimization targets using the Box–Behnken design (BBD) and response surface methodology (RSM). The total phenol content and antioxidant activity of *J. sabina* needle extract were also optimized. Under the optimal process conditions (ratio of material to liquid (RLM) 1:40, 90% methanol, and ultrasonic time 7 min), the podophyllotoxin extraction rate was 7.51 mg/g DW, the highest level reported for *Juniperus* spp. distributed in China. To evaluate its biological potential, the neuroprotective acetyl- and butyrylcholinease (AChE and BChE) inhibitory abilities were tested. The needle extract exhibited significant anti-butyrylcholinesterase activity (520.15 mg GALE/g extract), which correlated well with the high levels of podophyllotoxin and deoxypodophyllotoxin. This study shows the potential medicinal value of *J. sabina* needles.

## 1. Introduction

Podophyllotoxin is a natural lignan-like compound with significant antitumor properties. The anticancer drugs etoposide and teniposide were synthesized with it as precursor compounds and have emerged as first-line chemotherapeutic agents for the treatment of many cancers: lung, breast, ovarian, testicular, gastric, bladder, pancreatic, brain, and blood [1,2,3]. According to recent studies, etoposide was found to be targeted in the treatment of cytokine storms in patients with COVID-19 [4]. Owing to its significant clinical role, the extraction and biological study of podophyllotoxin and its analogues are important topics in current study.

The *Juniperus* genus is an alternative plant source of podophyllotoxin and its analogues [5]. In 1953, podophyllotoxin and deoxypodophyllotoxin were identified in the *Juniperus* genus [6]; today, they are found in many species: *Juniperus chinensis* [7,8], *Juniperus conferta* [7], *Juniperus davurica* [9], *Juniperus depressa* [7]; *Juniperus horizontalis* [8]; *Juniperus lucayana* [6]; *Juniperus scopulorum* [7,10], *Juniperus virginiana* L. [11,12,13], *Juniperus x media* [14], *Juniperus bermudiana* L. [5], *Juniperus horizontalis* [15], *Juniperus communis* L. [16], *Juniperus sabina* L. [17,18], and *Juniperus phoenicea* [19].

The needles of *J. sabina*, used in traditional Chinese medicine, are rich in podophyllotoxins of which 22 have been isolated (Table 1) [20]. In addition, the needles have significant beneficial physiological properties: antiviral, antirheumatic, antitussive, antitumor, antioxidant, hypolipidemic, neurotoxic, and immunosuppressive [21,22,23,24,25]. At present, there is an intent to develop industrial crops to provide drug precursors, and *J. sabina*, as a shrub representative of the *Juniperus* genus, should be considered for its suitability for cultivation and industrial application [26]. The rich bioactive ingredients and the advantages of resource application clearly show the potential for its needles in industries that produce pharmaceuticals and chemical functional products. Therefore, extracting high-efficiency biologically active compounds was a key step.

Consequently, this study used response surface methodology (RSM) to optimize the conditions for homogenization-assisted ultrasonic extraction of podophyllotoxin, deoxypodophyllotoxin and phenols for their antioxidant properties, which were further evaluated by an in vitro anticholinesterase assay. This study provides the possibility of using *J. sabina* as a potential source of podophyllotoxin and lays a foundation for the development of high value-added compounds from its needles.

## 2. Results and Discussion

### 2.1. Optimization of the Extraction Conditions

To obtain the best active extracts, the most influential variables were jointly optimized using the Box–Behnken design (BBD) and RSM. In the BBD, 17 experimental runs with five replicates (central point) were conducted. Based on the results (Table 2) for each dependent variable (*Y_n_*) after analyzing each of the 17 experimental runs, regression models were developed to determine the approximate and predicted functional relationships of the responses. Table 3 summarizes the significant regression coefficients (at 90% confidence interval) from the analysis of variance (ANOVA), coefficient of determination (R^2^), and the model. The prediction equations that demonstrated *Y_n_* using significant terms are

(1) $$Y_{TPC}=535.74+22.42X_{1}+81.53X_{2}-32.09X_{2}^{2}$$

(2) $$Y_{PPT}=7.76+0.21X_{1}-0.27X_{1}X_{3}+0.30X_{2}X_{3}+0.34X_{1}^{2}-0.20X_{2}^{2}$$

(3) $$Y_{DPT}=2.24+0.11X_{1}+0.09X_{2}+0.16X_{2}X_{3}+0.13X_{1}^{2}-0.20X_{2}^{2}$$

(4) $$Y_{E.yield}=313.32+37.58X_{2}-5.61X_{3}+9.98X_{2}X_{3}-25.86X_{2}^{2}$$

(5) $$\begin{array}{c} Y_{ABTS}=14106.40+1003.81X_{1}+926.46X_{2}-1028.40X_{3}-\\ 2213.13X_{2}^{2}-1179.76X_{3}^{2} \end{array}$$

(6) $$Y_{FRAP}=10572.0+926.46X_{2}-1617.19X_{2}^{2}$$

(7) $$Y_{DPPH}=399.38+975.24X_{2}+749.35X_{2}^{2}.$$

Analysis of the R^2^ values (0.9283–0.9812) showed high model accuracy for the extraction yield (E. yield), total phenol content (TPC), ferric reducing antioxidant power (FRAP), and DPPH, thus indicating a strong correlation between predicted and experimental values (Table 3). While the podophyllotoxin and deoxypodophyllotoxin content, and the ABTS models showed a low R^2^ value, they were all greater than 0.83 and indicated that the data deviation could be explained by each empirical model. Domínguez et al. [27] also applied the RSM to identify the optimum extraction conditions for the highly bioactive compounds of *Sambucus nigra* L.

#### 2.1.1. Total Phenol Content (TPC)

Phenolic compounds are secondary metabolites synthesized by plants under oxidative stress and one of the important adaptive mechanisms under various conditions of adversity [28,29]. TPC was quantified by the Folin–Ciocalteu reagent in the current study. As shown in Table 2, TPC under different experimental conditions (ECs) ranged from 388.39 to 611.27 mg GAE/g DW. The three best extractions were EC 2 (611.27 mg GAE/g DW), EC 7 (587.09 mg GAE/g DW) and EC 13 (596.02 mg GAE/g DW). Using our extraction parameters, very high concentrations of TPC were detected, higher than those obtained in other studies. Orhan et al. (2011) showed poor TPC values (68.43 and 122.67 mg GAE/g) for the aqueous and ethanolic extracts of *J. sabina* needles [30]. While analyzing 11 regions of *Juniperus drupacea*, the TPC of needles ranged from 2.69 to 53.82 mg GAE/g [31]. The TPC in *J. drupacea* berries was even higher (225.23 mg GAE/g) because phenolic acids accounted for more than 60% of total phenols [32].

**Figure 1:**
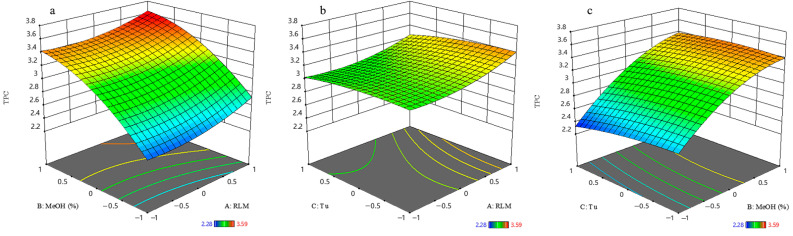
Response surface of total phenolic content: (**a**) as a function of ratio of material to liquid and percentage of methanol; (**b**) as a function of ratio of material to liquid and ultrasonic time; (**c**) as a function of percentage of methanol and ultrasonic time.

According to the multinomial Equation (1), the primary terms of the robust linear model (RLM) (*X*_1_) and methanol % (*X*_2_) had a positive effect on TPC retention. In particular, the variable methanol % exhibited a highly significant (*p* < 0.0001) influence on TPC yield. This fact suggests that most of the bioactive compounds in *J. sabina* needle extracts had low polarity. The response surface model of TPC in a function of the RLM (*X*_1_) and methanol % (*X*_2_) implied that both factors affected their recovery (Figure 1). It also indicated that intermediate values of RLM and high values of methanol % resulted in maximum recoveries (>546 mg GAE/g).

#### 2.1.2. Podophyllotoxin and Deoxypodophyllotoxin

**Figure 2:**
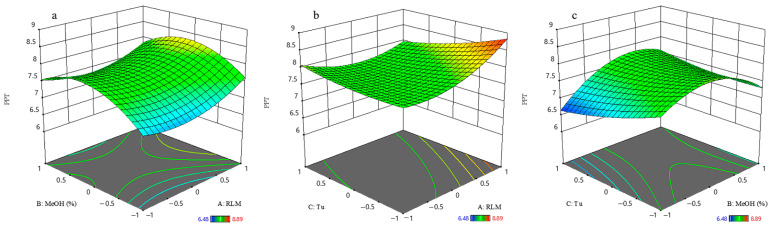
Response surface of podophyllotoxin content: (**a**) as a function of ratio of material to liquid and percentage of methanol; (**b**) as a function of ratio of material to liquid and ultrasonic time; (**c**) as a function of percentage of methanol and ultrasonic time.

The total amount of podophyllotoxin and deoxypodophyllotoxin was quantified by high-performance liquid chromatography (HPLC). Podophyllotoxin recovered from *J. sabina* needle extract ranged from 6.48 to 8.89 mg/g DW. The three most abundant runs were EC 6 (8.89 mg/g DW), EC 14 (8.08 mg/g DW) and EC 9 (8.03 mg/g DW) (Table 2). The three best extracts were all obtained by 80% methanol extraction. In addition, the ultrasonic time (*X*_3_) and RLM (*X*_1_) were mutually complementary (Figure 2, Equation (2)), and an appropriate increase in the ultrasonic time (*X*_3_) reduced the use of extraction solvent and thus the production cost. The podophyllotoxin content from the *J. sabina* needles was more than four times that reported by Renouard et al. [5]. Och et al. [7] analyzed 11 species (61 varieties) of *Juniperus* spp., including *J. sabina*, and found that podophyllotoxins ranged from 0.02 to 4.87 mg/g DW. Therefore, the extraction method in this study yielded the highest levels of podophyllotoxin reported so far from *J. sabina*.

Deoxypodophyllotoxin can be considered an alternative to podophyllotoxin, so optimization of extractions is equally important. The amount obtained from *J. sabina* needles under different experimental extraction conditions ranged from 1.75 to 2.49 mg/g DW. Three optimal extracts were obtained at 80–100% methanol, 5–10 min ultrasonic time, and a RLM of 1:30–1:40 (Supplementary Material Figure S1), i.e., EC 14 (2.40 mg/g DW), EC 2 (2.46 mg/g DW) and EC 6 (2.49 mg/g DW) (Table 2). From Equation (3), both the primary and secondary terms of RLM (*X*_1_) correlated positively with deoxypodophyllotoxin yield. In addition, methanol % (*X*_2_) and ultrasonic time (*X*_3_) might have had a synergistic effect on the yield. The above results showed that the main influencing yield factor was RLM (*X*_1_), with methanol % (*X*_2_) and ultrasonic time (*X*_3_) playing a secondary role. 

#### 2.1.3. Extraction Yield (E. Yield) 

The E. yield obtained under different experimental conditions (EC) ranged from 222.10 to 329.15 mg/g DW, in which the three ECs with the highest yield were EC 2 (322.30 mg/g DW), EC 13 (324.55 mg/g DW) and EC 7 (329.15 mg/g DW), as shown in Table 2. These three best ECs were consistent with those for TPC, indicating that the TPC value contributed the most to the extract. However, there was no significant correlation between the value of the E. yield and that of the podophyllotoxins. Overall, response surfaces can provide an efficient way to achieve the desired optimization goals for extracts with different properties [27].

The response surface model of E. yield as a function of methanol % (*X*_2_) and ultrasonic time (*X*_3_) showed that methanol % (*X*_2_) significantly affected their recovery (Supplementary Material Figure S2). The E. yield increased by 48% when methanol % (*X*_2_) was high compared to low. In addition, Equation (4) confirmed these considerations, where the linear term of methanol % (*X*_2_) and the product term of methanol % and RLM (*X*_2×3_) both had a positive effect on the recovery of E. yield (Table 2).

#### 2.1.4. Antioxidant Activity In Vitro

Extracts of *J. sabina* needles showed significant in vitro antioxidant activity [30]. The influence of extraction conditions on antioxidant capacity was determined using three antioxidant assays: ABTS, FRAP and DPPH. As shown in Table 2, the highest antioxidant activity was detected at EC 4, EC 17 and EC 7 for ABTS (15,922.9 µmol Trolox/100 g DW), FRAP (11,702.4 µmol Trolox/100 g DW) and DPPH (2360.2 µmol Trolox/100 g DW). The above results showed that the extracts with high antioxidant activity were more likely to be obtained when methanol % (*X*_2_) was around 80%. In addition, the antioxidant activity of *J. sabina* needle extract was found to be significantly better than that of common sources of antioxidants, such as *Rubus rosaefolius* or *Sambucus nigra* L. [27,33].

Considering the regression coefficients (Table 3), the quadratic terms of methanol % (*X*_2_) all significantly affected antioxidant activity (*p* < 0.01), having a negative effect on ABTS and FRAP capacity (Supplementary Material Figures S3 and S4). The variation pattern of Supplementary Material Figure S5a,c showed that the FRAP values of the extracts all started to decrease as the methanol % increased to about 80%. Piwowarska and González-Alvarez [34] analyzed the antioxidant capacity of forestry biomass extracts on the dependence of solvent concentration and obtained similar results. In addition, both the liner and quadratic terms showed a positive effect on DPPH capacity (Equation (7)), and the DPPH values of the extracts increased with methanol % (Supplementary Material Figure S5a,c). Thoo et al. [35] reported that the DPPH in *Morinda citrifolia* extracts increased with ethanol concentration.

In summary, the TPC and antioxidant capacity of *J. sabina* needles were significantly affected by the methanol % as shown in the response surface plots of all dependent variables. There were complex influences between the active compounds and their bioactive properties as well as the potential pharmacodynamic effects among different molecules in the extracts [36,37]. Simultaneous optimization, therefore, could maximize the content of podophyllotoxins, phenols and their antioxidant activity.

#### 2.1.5. Process Optimization and Model Validation

**Figure 3:**
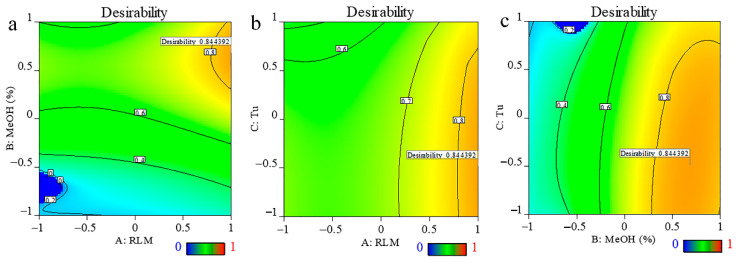
Desirability surface plot: (**a**) as a function of ratio of material to liquid (RLM) and percentage of methanol; (**b**) as a function of RLM and ultrasonic time; (**c**) as a function of percentage of methanol and ultra-sonic time.

The aim of the optimization was to determine the extraction conditions that would provide the greatest amount of podophyllotoxin and deoxypodophyllotoxin. At the same time, other targets (TPC, E. yield, and antioxidant activity) were set to be greater than the range above the median. Figure 3 showed the contour plot as a function of RLM, methanol % and ultrasonic time. The parameters selected as optimal showed desirability values > 0.70, which was within an acceptable range. The optimal extraction conditions were RLM 1:40, 93.7% methanol and ultrasonic time 7.575 min (supplementary Material Table S2). To test the reliability of the predicted results and to take into account the convenience of practical operation, the optimal extraction conditions were modified to RLM 1:40, 90% methanol, and ultrasonic time to 7 min. The relative standard deviations (RSDs) of all variables (Table 4) showed that the predicted values for all groups were very similar to the experimental results, except for DPPH, which had an RSD value (22.52 > 10). The suitability of the response surface methodology model for quantitative predictions was verified by satisfactory agreement between the predicted and measured values. These findings also justified the selection of the Box–Behnken design, which had been demonstrated to be accurate and reliable for predicting the content of podophyllotoxins, TPC, E. yield, and antioxidant capacities of the extracts.

### 2.2. Anticholinesterase Activity

Inhibition of acetylcholinesterase (AChE) and butyrylcholinesterase (BChE) is one of the important strategies for combatting Alzheimer’s disease (AD) [38]. Natural products are an abundant source of novel bioactive compounds [39], and in this study, extracts of *J. sabina* needles obtained under different conditions were analyzed for AChE and BChE as potential natural enzyme inhibitors, with values expressed as galanthamine equivalents. As shown in Table 5, the inhibitory activity values of the extracts against AChE and BChE were 0.96–28.79 mg GALE/g and 37.67–520.15 mg GALE/g, respectively. The results showed that the needle extracts had good anti-BChE activity. Orhan et al. [30] analyzed the anticholinesterase activity of *Juniperus* leaf extracts and similarly found that the BChE inhibitory activity was better than that of AChE. Ballard et al. [40] demonstrated a significant inhibitory effect of *J. sabina* needle extracts on BChE, presumably related to the presence of this enzyme as a major cholinesterase in the brains of patients with advanced AD.

**Figure 4:**
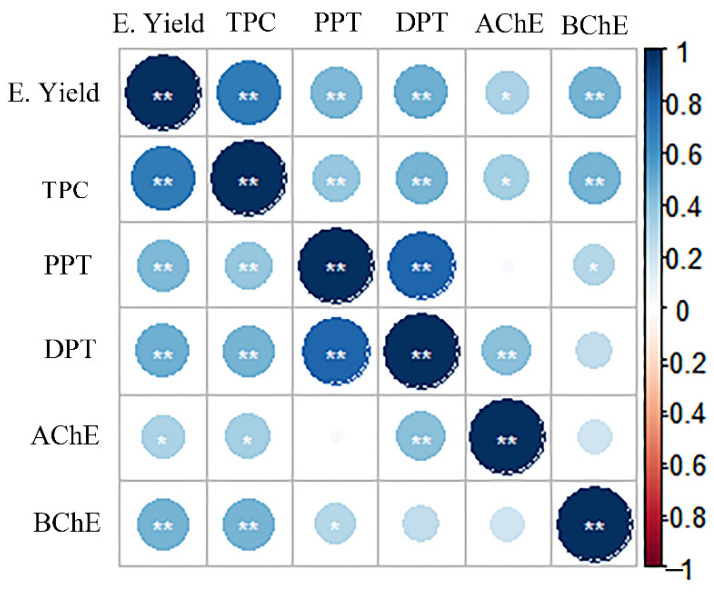
Relationship between bioactive compounds and biological activities in the needles of *J. sabina.* “*”: *p* < 0.05; “**”: 0.01 < *p* < 0.05; E. yield: the extraction yield; TPC: total phenol content; PPT: podophyllotoxin; DPT: deoxypodophyllotoxin; AChE/BChE: the anti-cholinesterase activity values of *J. sabina* extracts.

A correlation analysis between extract content and anticholinesterase activity was performed to further understand the pharmacodynamic properties of the extracts tested. The results showed (Figure 4) that the anti-AChE activity of the needle extracts showed a significant correlation (*p* < 0.05) with E. yield and TPC, while it showed a highly significant correlation (*p* < 0.01) with deoxypodophyllotoxin. The values of E. yield and TPC both showed a highly significant correlation (*p* < 0.01) with that of anti-BChE activity, and podophyllotoxin content also suggested a significant correlation (*p* < 0.05). From a statistical point of view, podophyllotoxins and the phenolics in the *J. sabina* needle extracts may contribute to cholinesterase inhibition. Therefore, their enzyme inhibitory properties may have a phytochemical interaction; that is, phenols and podophyllotoxins may have a synergistic effect in inhibiting cholinesterase activity, but the specific mechanism needs further analysis.

## 3. Material and Methods

### 3.1. Chemicals

For extractions and solutions, ultrapure water was obtained by a Milli-Q system Millipore (Bedford, MA, USA) and absolute ethanol was purchased from Sigma-Aldrich (St. Louis, MO, USA). The 7% sodium carbonate (Na_2_CO_3_) solution was obtained by dissolving 7 g of powder in ultrapure water and making up to 100 mL.

The following reagents were supplied by specified suppliers: Gallic acid, Folin reagent, ferric chloride (FeCl_3)_, dimethyl sulfoxide (DMSO) and Na_2_CO_3_ were purchased from Aladdin (Aladdin Biochemical Technology Co., Ltd., Shanghai, China). Acetylcholinesterase (Electric eel), butyrylcholinesterase (Horse serum), acetylthiocholine iodide, S-butyrylthiocholine iodide, 5,5’-dithio bis-(2-nitrobenzoic acid), and Tris (tri (hydroxymethyl) aminomethane) were purchased from Macklin (Macklin Biochemical Co., Ltd., Shanghai, China). DPPH (1,1-diphenyl-2 picrylhydrazyl radical), ABTS (2,2-azinobis (3-ethylbenzothiazoline-6-sulphonic acid) radical cation), TPTZ (2,4,6-tri(2-pyridyl)-s-triazine), Trolox (6-hydroxy-2,5,7,8-tetramethylchroman-2-carboxylic acid), acetic acid, podophyllotoxin, and deoxypodophyllotoxin were purchased from Sigma-Aldrich (St. Louis, MO, USA).

### 3.2. Plant Material 

The *J. sabina* needles used in this experiment were collected in October 2020 from Expo Park of Northwest Agriculture and Forestry University (Yangling District, Shaanxi Province, China, 34°18′3″ N 108°2′56″ E). Healthy growing needles were selected, freeze-dried, crushed, passed through a 40-mesh sieve, sealed and packed for storage in a dry place for later use.

### 3.3. HPLC Analysis

Podophyllotoxin and deoxypodophyllotoxin content were determined according to Renouard et al. [5] with minor modifications. Liquid phase analysis was performed by HPLC (Agilent 1260, Agilent Technologies Inc., Santa Clara, CA, USA) with a C18 (5 μm, 4.6 × 250 mm; Agilent Technologies Inc., Santa Clara, CA, USA) column. The mobile phases were methanol (B) and 0.2% formic acid-water solution (D) with the following gradients: 0–10 min, 10–30% B; 10–20 min, 30–55% B; 20–35 min, 55–80% B; 35–40 min, 80–84% B; 40–45 min, 84–100% B; 45–50 min, 100% B; 50–52 min, 100–10% B; 52–56 min, 10% B. The flow rate was 0.8 mL/min; the column temperature was 35 ℃; and the injection volume was 20 μL. Podophyllotoxin and deoxypodophyllotoxin standards were purchased from Yuanye (Yuanye Bio-Technology Co., Ltd., Shanghai, China). The standard solutions were prepared in methanol with an initial concentration of 1 mg/mL and the standard curve was drawn after gradient dilution (podophyllotoxin: y = 9736x + 9.1552, R^2^ = 0.9946; deoxypodophyllotoxin: y = 22,774x + 5.4356, R^2^ = 0.9995). The peak area (y) was used to calculate the content of the target compounds.

### 3.4. Extraction of J. sabina

The extract of *J. sabina* needles was obtained by homogenization-assisted ultrasonic extraction. Needle powder (2 g) was weighted into a 100 mL Erlenmeyer flask and 40–80 mL of extraction solvent was added (for relevant extraction conditions, see Supplementary Material Table S1). This mixture was then sonicated at 100 kHz for 5–15 min in an ultrasonic bath (Branson, Mod. 8510E-DTH, Danbury, CT, USA). The mixture was extracted for 1.5 h at 55 °C under stirring in a water bath (HSJ-4, Jiangsu Science Analysis Instrument Co., Ltd., Changzhou, China). The extract mixture was centrifuged at 10,000× *g* for 10 min and the supernatant was drawn off, concentrated under reduced pressure and then evaporated to a constant weight.

E. Yield(mg/g DW) = mass of extraction (mg)/dry weight of material (g)

E. Yield is the extraction yield of *J. sabina*; DW refers to the dry weight of material; mass of extraction refers to the mass of the extract dried to constant weight.

### 3.5. Experimental Design

The effects of the feed-to-liquid ratio (*X*_1_; g/mL), methanol concentration (MeOH; *X*_2_; %), ultrasonic time (T_U_; *X*_3_; min) on the extraction yield (E. yield), antioxidant activities, and total phenol, podophyllotoxin and deoxypodophyllotoxin content were investigated (Supplementary Material Table S1). Response surface experiments were designed and optimized using the (BBD) approach with three replicates at the center point. The experimental data were fitted to the following second-order polynomial model equation.

(8) $$Y_{j}=\beta_{0}+\sum_{i=1}^{3}\beta_{i}X_{i}+\sum \sum_{i<j=1}^{3}\beta_{ij}X_{i}\beta_{i}X_{ij}+\sum_{i=j}^{3}\beta_{ii}X_{ii}$$

*Y_j_* is the dependent (response) variable; *X_i_*, *X_ij_* and *X_ii_* were the independent variables; and *β*_0_, *β_i_*, *β_ij_* and *β_ii_* were the regression coefficients. The adequacy of the model was determined by assessing the misfit, coefficient of determination (R^2^) and the F-test values from the ANOVA.

### 3.6. Determine Optimal Conditions and Validate the Model

Multi-response surface optimization was used to maximize the selected response variables simultaneously. Selection criteria were based on maximizing podophyllotoxin and deoxypodophyllotoxin content, taking into account the optimization of TPC, E. yield and antioxidant activity obtained by DPPH, FRAP and ABTS. Optimal extraction conditions were estimated using the response expectancy analysis function of Design-Expert software. Model validation was performed by conducting experiments under optimal extraction conditions, and the values predicted by each model were compared with the experimental data. The similarity between the experimental and predicted data was calculated using relative standard deviation (*RSD*):

(9) $$RSD\;\%=\frac{Standard\;deviation\;between\;predicted\;and\;experimented\;values}{Mean\;valves\;between\;predicted\;and\;experimental\;valves}\times 100$$

At *RSD* % < 10, the resulting data were considered similar, and the results were analyzed and optimized for all response conditions. 

### 3.7. Determination of Total Phenol Content (TPC)

In this experiment, the TPC of *J. sabina* needle extract was determined by the Folin-Ciocalteu colorimetric method [41,42]. After pipetting 200 μL of extract (2 mg/mL) into a test tube, 2 mL of Folin–Ciocalteu reagent was added. The mixture was shaken well and left to react for 6 min before adding 2 mL of 7% Na_2_CO_3_ solution (mass to volume ratio) and reading the absorbance at 760 nm on an ultraviolet spectrophotometer (UV-1800, Shimadzu Manufacturing Co., Kyoto, Japan). Three parallel sets were made for all the experiments.

### 3.8. Evaluation of In Vitro Biological Activity

Extracts of *J. sabina* needles were prepared in a methanol solution to 32 mg/mL mother liquor, stored at 4 °C to be ready to use. The related activity assay was completed within three days.

#### 3.8.1. Antioxidant Activity In Vitro

The antioxidant activity was assessed using several different in vitro assays (ABTS, FRAP, and DPPH) according to the procedures described by Peng et al. [43]. The results were expressed as Trolox equivalents (µmol Trolox/100 g DW).

#### 3.8.2. Cholinesterase Inhibitory Activity

The cholinesterase inhibitory activity assay was determined using the modified spectrophotometric method of Ellman et al. [44]. A 96-well plate was prepared by adding 140 µL of phosphate buffer, 20 µL of AChE or BChE and 20 µL of *J. sabina* needle extract (500 µg/mL), mixed well and incubated for 15 min at 25 °C. After 30 min, the absorbance values of the solutions were measured at 412 nm using an enzyme marker. DMSO was used as a negative control; galanthamine was used as a positive control; and the blank group had 20 µL of phosphate buffer added. The first concentration of galanthamine was 640 µg/mL and used for standard curve drawing after gradient dilution. Three parallel experiments were done for each group and average values were taken. The cholinesterase inhibition rate was then calculated according to the following equation.

Inhibition rate (%) = (Blank group − Experimental group)/Blank group × 100%

Experimental group: absorbance value of compound to be measured; blank group: phosphate buffer instead of absorbance value of compound to be measured.

### 3.9. Data Analysis

The assays described were performed in triplicate for all experiments, and the results were expressed as mean ± standard deviation (SD). All response surface methodology (RSM) data were statistically analyzed to determine the significant parameters and the interaction between each variable. Specifically, response surface analysis was performed using Design-Expert11 (Stat-Ease, Inc., Minneapolis, MN, USA). Data variance significance processing and analysis were performed using Statistics V8.0 (Statsoft Inc., Tulsa, OK., USA) and SPSS 13.0 (SPSS Inc., Chicago, IL., USA). In all cases, *p* < 0.05 indicated significance.

## 4. Conclusions

This study determined the optimal process conditions (RLM 1:40, 90% methanol and ultrasonication time 7 min) for obtaining *J. sabina* needle extract with the highest content of podophyllotoxin (7.51 mg/g DW), high TPC, and satisfactory antioxidant activity. The biological activity indices were added to the RSM models for optimization, bringing them more in line with the complexity of natural drug applications and offering more economical and effective optimization conditions. In addition, the results of the excellent anticholinesterase activity of the extracts was interesting, and the significant positive effects of podophyllotoxin and deoxypodophyllotoxin on it were also observed. The study suggests that *J. sabina* could be an economical source of raw material for podophyllotoxins, antioxidants and Alzheimer’s inhibition.

## Figures and Tables

**Table 1:** Podophyllotoxin (1) and its analogues (2–22) isolated from *J. sabina*.

NO.	Compound Name	Structure	
1	Podophyllotoxin	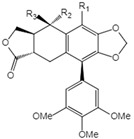	1 R_1_=R_3_=H, R_2_=OH 2 R_1_=R_2_=H, R_3_=OH 3 R_1_=R_2_=H, R_3_=OAc 4 R_1_=R_2_=R_3_=H 5 R_1_=OMe, R_2_=OH, R_3_=H 6 R_1_=OMe, R_2_=R_3_=H
2	Epipodophyllotoxin		
3	Epipodophyllotoxin acetate		
4	Deoxypodophyllotoxin		
5	2′-Methoxy-podophyllotoxin		
6	β-Methylpeltatin A		
7	Deoxy-picropodophyllotoxin	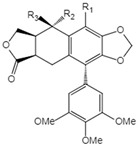	7 R_1_=R_2_=R_3_=H 8 R_1_=R_3_=H, R_2_=OH 9 R_1_=R_3_=H, R_2_=OAc 10 R_1_=R_2_=H, R_3_=OH 11 R_1_=R_2_=H, R_3_=OAc 12 R_1_=OMe, R_2_=OH, R_3_=H 13 R_1_=OMe, R_2_=H, R_3_=OH
8	Picropodophyllotoxin		
9	Picropodophyllotoxin acetate		
10	Epipicropodophyllotoxin		
11	Epipicropodophyllotoxin acetate		
12	2′-Methoxy-picropodophyllotoxin		
13	2′-Methoxy-epipicropodophyllotoxin		
14	Dehydropodophyllotoxin	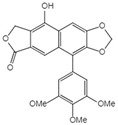	
15	Acetyl epipodophyllotoxin	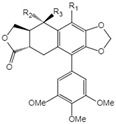	15 R_1_=R_2_=H, R_3_=OAc 16 R_1_=O, R_2_=R_3_=H 17 R_1_=H, R_3_=O
16	β-peltatin-A methylether		
17	Podophyllotoxone		
18	Acetyl epipicropodophyllotoxin	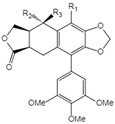	18 R_1_=R_2_=H, R_3_=OAc 19 R_1_=OMe, R_2_= H, R_3_=OH 20 R_1_=H, R_2_=R_3_=O
19	β-peltatin-B methylether		
20	Picropodophyllotoxone		
21	Junaphthoic acid	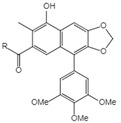	21 R=OH 22 R=OAc
22	4-Acetyl junaphtholic acid		

**Table 2:** Box–Behnken experimental design (expressed as independent variables) and experimental results obtained for dependent variables.

EC	RLM	Methanol (%)	T_U_ (min)	TPC	PPT	DPT	E. Yield	ABTS	FRAP	DPPH
	Independent Variables			Dependent Variables						
	*x*_1_	*x*_2_	*x*_3_	*y*_1_	*y*_2_	*y*_3_	*y*_4_	*y*_5_	*y*_6_	*y*_7_
1	1:40 (1)	60 (−1)	10 (0)	483.27 ± 40.96	7.61 ± 0.32	2.20 ± 0.07	253.85 ± 9.25	12,462.3 ± 602.4	7880.3 ± 158.6	194.0 ± 69.0
2	1:40 (1)	100 (1)	10 (0)	611.27 ± 91.04	7.61 ± 0.33	2.46 ± 0.11	322.30 ± 3.60	14,606.7 ± 107.4	10,584.9 ± 284.5	2129.5 ± 88.4
3	1:30 (0)	80 (0)	10 (0)	546.53 ± 44.48	7.82 ± 0.09	2.33 ± 0.09	316.60 ± 3.90	15,454.3 ± 612.0	10,139.1 ± 85.5	645.7 ± 145.3
4	1:30 (0)	80 (0)	10 (0)	537.60 ± 44.30	7.91 ± 0.14	2.20 ± 0.06	314.60 ± 7.00	15,922.9 ± 285.2	10,855.9 ± 383.9	444.8 ± 6.0
5	1:20 (−1)	100 (1)	10 (0)	546.76 ± 88.62	7.57 ± 0.28	2.03 ± 0.07	318.50 ± 2.60	12,281.6 ± 721.9	9880.6 ± 262.1	2086.3 ± 271.3
6	1:40 (1)	80 (0)	5 (−1)	582.62 ± 36.37	8.89 ± 0.46	2.49 ± 0.13	307.50 ± 3.90	14,940.4 ± 926.5	11,009.4 ± 713.0	324.4 ± 158.9
7	1:30 (0)	100 (1)	5 (−1)	587.09 ± 68.29	7.49 ± 0.57	2.01 ± 0.16	329.15 ± 2.75	13,477.8 ± 1040.0	11,155.0 ± 316.3	2360.2 ± 62.4
8	1:30 (0)	80 (0)	10 (0)	515.27 ± 46.66	7.41 ± 0.65	2.02 ± 0.21	314.55 ± 0.85	13,291.0 ± 910.0	9623.9 ± 664.1	311.0 ± 105.8
9	1:20 (−1)	80 (0)	15 (1)	515.27 ± 35.77	8.03 ± 0.22	2.23 ± 0.05	305.05 ± 1.05	10,652.7 ± 305.0	9873.9 ± 384.6	280.8 ± 111.4
10	1:30 (0)	60 (−1)	5 (−1)	412.95 ± 29.03	7.51 ± 0.10	2.12 ± 0.09	266.60 ± 1.10	9903.6 ± 376.8	7823.2 ± 112.6	138.0 ± 56.1
11	1:20 (−1)	80 (0)	5 (−1)	538.72 ± 45.32	7.70 ± 0.31	2.37 ± 0.09	307.65 ± 6.45	14,467.8 ± 488.1	11,343.7 ± 754.0	192.1 ± 121.2
12	1:20 (−1)	60 (−1)	10 (0)	422.25 ± 25.22	7.28 ± 0.19	2.01 ± 0.06	251.30 ± 0.80	11,013.7 ± 365.2	7368.7 ± 86.5	122.7 ± 18.1
13	1:30 (0)	100 (1)	15 (1)	596.02 ± 99.99	7.64 ± 0.57	2.28 ± 0.26	324.55 ± 3.25	9949.0 ± 134.1	11,657.2 ± 221.5	1689.3 ± 41.1
14	1:30 (0)	80 (0)	10 (0)	543.18 ± 50.87	8.08 ± 0.26	2.40 ± 0.15	308.80 ± 2.20	13,122.1 ± 234.7	11,144.7 ± 86.5	301.9 ± 143.8
15	1:30 (0)	60 (−1)	15 (1)	388.39 ± 24.49	6.48 ± 0.12	1.75 ± 0.11	222.10 ± 1.30	9523.8 ± 183.6	6930.0 ± 135.6	8.7 ± 1.2
16	1:30 (0)	80 (0)	10 (0)	536.11 ± 55.07	7.57 ± 0.51	2.24 ± 0.22	319.50 ± 2.90	12,741.9 ± 353.5	11,096.4 ± 513.9	293.5 ± 212.9
17	1:40 (1)	80 (0)	15 (1)	543.18 ± 43.06	8.16 ± 0.03	2.38 ± 0.00	314.30 ± 3.90	14,436.9 ± 557.3	11,702.4 ± 347.5	339.1 ± 8.7

EC: Experimental conditions; E. Yield (mg/g DW); TPC (mg GAE/g DW); PPT (mg/g DW); DPT (mg/g DW); ABTS (μmol trolox/100 g DW); FRAP (μmol trolox/100 g DW); DPPH (μmol trolox/100 g DW); DW: dry weight of material.

**Table 3:** Regression coefficients and statistical parameters measuring the correlation and significance of the models.

	TPC	PPT	DPT	E. Yield	ABTS	FRAP	DPPH
	*y*_2_	*y*_3_	*y*_4_	*y*_1_	*y*_5_	*y*_6_	*y*_7_
β_0_	535.74	7.76	2.24	313.32	14,106.40	10,572.00	399.38
β_1_	22.42 ^b^	0.21 ^c^	0.11 ^b^	1.93	1003.81 ^b^	338.76	38.14
β_2_	81.53 ^a^	0.18	0.09 ^c^	37.58 ^a^	926.46 ^c^	1659.44 ^a^	975.24 ^a^
β_3_	−9.81	−0.16	−0.04	−5.61 ^c^	−1028.40 ^b^	−145.98	−87.10
β_12_	−3.63	−0.07	0.06	0.31	219.13	48.18	−7.03
β_13_	−4.00	−0.27 ^c^	0.01	2.35	827.90	540.70	−18.50
β_23_	8.37	0.30 ^c^	0.16 ^b^	9.98 ^b^	−787.25	348.85	−135.40
β_11_	16.74	0.34 ^b^	0.13 ^c^	−2.83	697.77	−26.19	−15.60
β_22_	−32.09 ^b^	−0.58 ^a^	−0.20 ^b^	−25.86 ^a^	−2213.13 ^a^	−1617.19 ^a^	749.35 ^a^
β_33_	−7.53	0.10	−	−	−1179.76 ^c^	436.54	−99.68
R^2^	0.9612	0.8642	0.8319	0.9627	0.8687	0.9283	0.9812

^a^ Significant coefficients at 99% confidence interval (CI). ^b^ Significant coefficients at 95% CI. ^c^ Significant coefficients at 90% CI.

**Table 4:** Predicted and experimental values under optimum conditions resulting from the simultaneous optimization of the eight responses.

	PPT	DPT	TPC	E. Yield	ABTS	FRAP	DPPH
Predicted value	8.24	2.45	464.90	324.52	15,638.40	11,092.20	1506.86
Experimental value	7.51	2.35	540.1	309.31	17,242.14	10,963.49	2211.19
Std Dev	0.52	0.07	53.11	10.76	1134.01	91.01	2211.19
RSD (%)	6.88	3.15	9.84	3.48	6.58	0.83	22.52

**Table 5:** Anticholinesterase activity of *J. sabina* needle extracts under different extraction conditions.

EC	AChE (mg GALE/g Extract)	BChE (mg GALE/g Extract)
JS-1	9.64 ± 2.69	63.95 ± 10.37
JS-2	28.79 ± 4.14	282.99 ± 47.85
JS-3	2.61 ± 1.28	91.18 ± 14.77
JS-4	2.29 ± 1.17	86.36 ± 13.99
JS-5	2.06 ± 1.09	351.8 ± 60.4
JS-6	8.43 ± 2.53	284.77 ± 48.17
JS-7	9.26 ± 2.64	361.18 ± 160.67
JS-8	3.86 ± 1.64	434.77 ± 80.93
JS-9	1.99 ± 1.06	254.69 ± 100.49
JS-10	0.96 ± 0.63	173.4 ± 28.55
JS-11	8.28 ± 2.5	384.58 ± 66.48
JS-12	2.74 ± 1.32	125.96 ± 20.51
JS-13	17.01 ± 3.4	242.55 ± 40.62
JS-14	18.48 ± 3.5	520.15 ± 92.2
JS-15	5.02 ± 1.91	37.67 ± 6.21
JS-16	13.5 ± 3.11	109.39 ± 17.76
JS-17	7.07 ± 2.31	185.64 ± 30.65
JS-Y	16.78 ± 4.61	208.79 ± 41.66

EC: Experimental condition; JS-1~17: Extracts of *J. sabina* needles under different experimental conditions; JS-Y: Extracts of *J. sabina* needles under ideal extraction condition. Test extract concentration: 500 µg/mL DW extract.

## Data Availability

Not applicable.

## Supplementary Materials

The following supporting information can be downloaded at: https://www.mdpi.com/article/10.3390/ijms231810205/s1.
