# Supplementary material for: Juniperus sabina L. as a Source of Podophyllotoxins: Extraction Optimization and Anticholinesterase Activities
Source: Int J Mol Sci. 2022 Sep 6;23(18):10205. doi: 10.3390/ijms231810205 (PMC9499582; doi:10.3390/ijms231810205)
Supplement: Supplementary file 1 [file ijms-23-10205-s001.zip › ijms-1886393-supplementary.pdf]

Supplementary Material-Tables

Supplementary material Table S1. Experimental variables involved in the study.

Variable	Definition and Units	Nomenclature	Value/range
Fixed	Extraction Temperature (ºC)		55 ºC
	Extraction time (hours)		1.5 h
	Shaking speed (rpm)		150 rpm
	Ultrasonic power		100 KHz
Independent	Ratio of material to liquid (g/mL)	RLM (x_1)	1:20-1:40
	Methanol concentration (% v/v)	Methanol % (x_2)	60-100
	Ultrasonic time (min)	T_U (x_3)	5-15
Dependent	Total phenolic content (mg GAE/g DW)	TPC (y_1)	
	Podophyllotoxin content (mg/g DW)	PPT (y_2)	
	Deoxypodophyllotoxin content (mg/g DW)	DPT (y_3)	
	Extraction yield (mg/g DW)	E. yield (y_4)	
	ABTS (μmol trolox /100g DW)	ABTS (y_5)	
	FRAP (μmol trolox /100g DW)	FRAP (y_6)	
	DPPH (μmol trolox /100g DW)	DPPH (y_7)	

DW: Dry weight.

Supplementary material Table S2. Predicted and experimental values under optimum conditions resulting from the simultaneous optimization of the seven responses considered.

Factor	Independent variables	Solution	Optimal extraction condition	Desirability
A	RLM	1	1:40	
B	MeOH (%)	0.685	93.7	0.867
C	Tu（min）	-0.485	7.575	

Supplementary Material-Figues

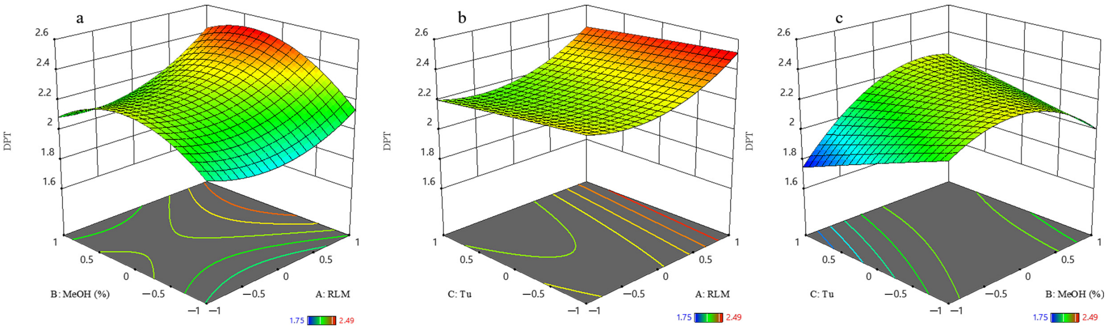

Supplementary material Figure S1. Response surface of deoxypodophyllotoxin content (a) as a function of ratio of material to liquid and percentage of methanol; (b) as a function of ratio of material to liquid and ultrasonic time; (c) as a function of percentage of methanol and ultrasonic time.

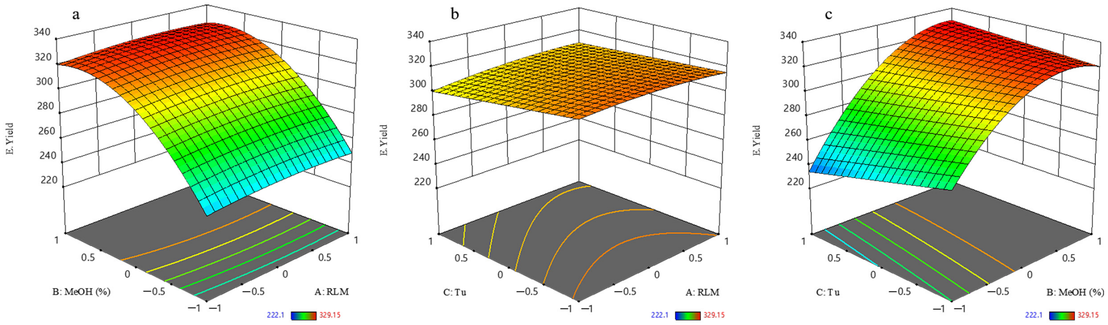

Supplementary material Figure S2. Response surface of extraction yield: (a) as a function of ratio of material to liquid and percentage of methanol; (b) as a function of ratio of material to liquid and ultrasonic time; (c) as a function of percentage of methanol and ultrasonic time.

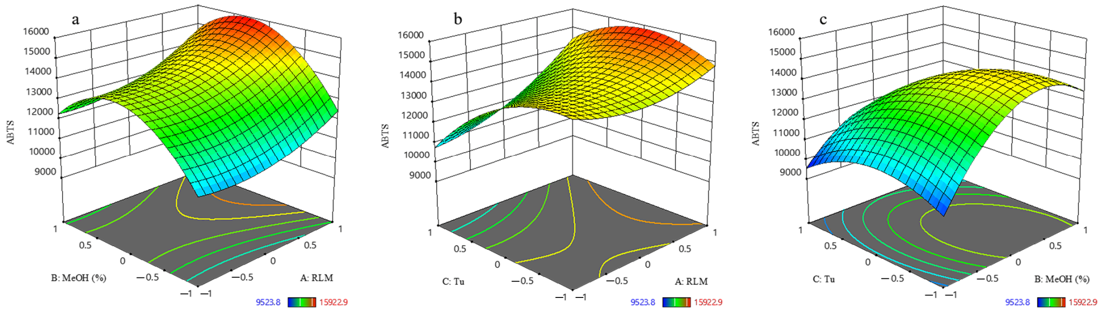

Supplementary material Figure S3. Response surface of ABTS capacity (a) as a

function of ratio of material to liquid and percentage of methanol; (b) as a function of ratio of material to liquid and ultrasonic time; (c) as a function of percentage of methanol and ultrasonic time.

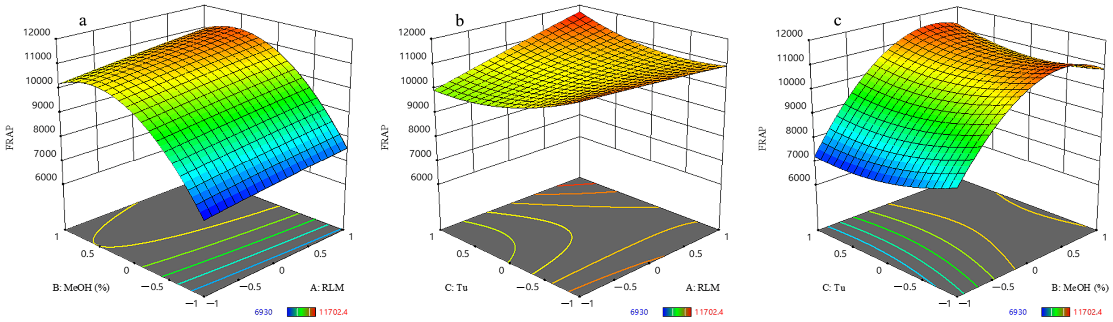

Supplementary material Figure S4. Response surface of FRAP capacity (a) as a function of ratio of material to liquid and percentage of methanol; (b) as a function of ratio of material to liquid and ultrasonic time; (c) as a function of percentage of methanol and ultrasonic time.

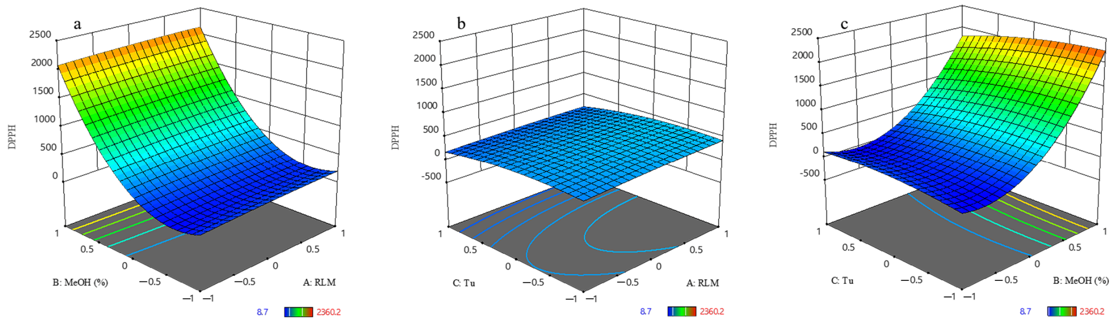

Supplementary material Figure S5. Response surface of DPPH activity (a) as a function of ratio of material to liquid and percentage of methanol; (b) as a function of ratio of material to liquid and ultrasonic time; (c) as a function of percentage of methanol and ultrasonic time.
